# Ossification of ungular cartilages in front feet of cold-blooded trotters - a clinical radiographic evaluation of development over time

**DOI:** 10.1186/s13028-014-0073-z

**Published:** 2014-10-30

**Authors:** Ulf O Hedenström, Ulf Olsson, Arne W Holm, Ove S Wattle

**Affiliations:** National Equine Education Centre Wången, SE-83593 Alsen, Sweden; Department of Economics, Swedish University of Agricultural Sciences, SE-75007 Uppsala, Sweden; Ökern vn 188, 0580 Oslo, Norway; Section for Large Animal Medicine and Surgery, Department of Clinical Sciences, Swedish University of Agricultural Sciences, S-75007 Uppsala, Sweden

**Keywords:** Equine, Horse, Collateral cartilages, Sidebones, Side bones, Distal phalanx, Radiology, Statistical methods

## Abstract

**Background:**

It has not yet been shown that ossification of ungular cartilages (OUC) is a pathological condition. Beside heredity, factors such as sex, age, repeated concussion, local trauma, hoof and body size have been suggested as contributing factors for OUC development. By comparing radiographs of front hooves from cold-blooded trotters with different age we wanted to evaluate when development of OUC in cold-blooded trotters occurs and if and when it stabilizes in relation to age and workload. Diagnosis and grading of OUC were based on radiological field examinations of 649 Swedish and Norwegian cold-blooded trotters’ front hooves. A hundred and forty-seven of the horses were re-examined 3-13 years (mean age 9, median 8 years) after the first occasion. All radiographs were evaluated blind, using two different grading systems for OUC. Work load, in form of number of races completed, and body size score were collected from official data. Four statistical ordinal regression models were used, compared and evaluated.

**Results:**

We identified a breakpoint at 2.8 ± 0.38 years of age when ossification ends and proposed a simpler grading system with more consistent results. There was no significant correlation between body size and grade of OUC. Comparison of different statistical methods for evaluation of ordinal data revealed a piecewise linear regression model as most suitable.

**Conclusions:**

Individuals with OUC developed this condition during the stage of life when their hooves develop in size. Results from this study can assist equine practitioners when examining and for understanding this condition in their clinical work and is also beneficial for the Scandinavian equine industry when devising breeding programs.

## Background

**Figure 1:**
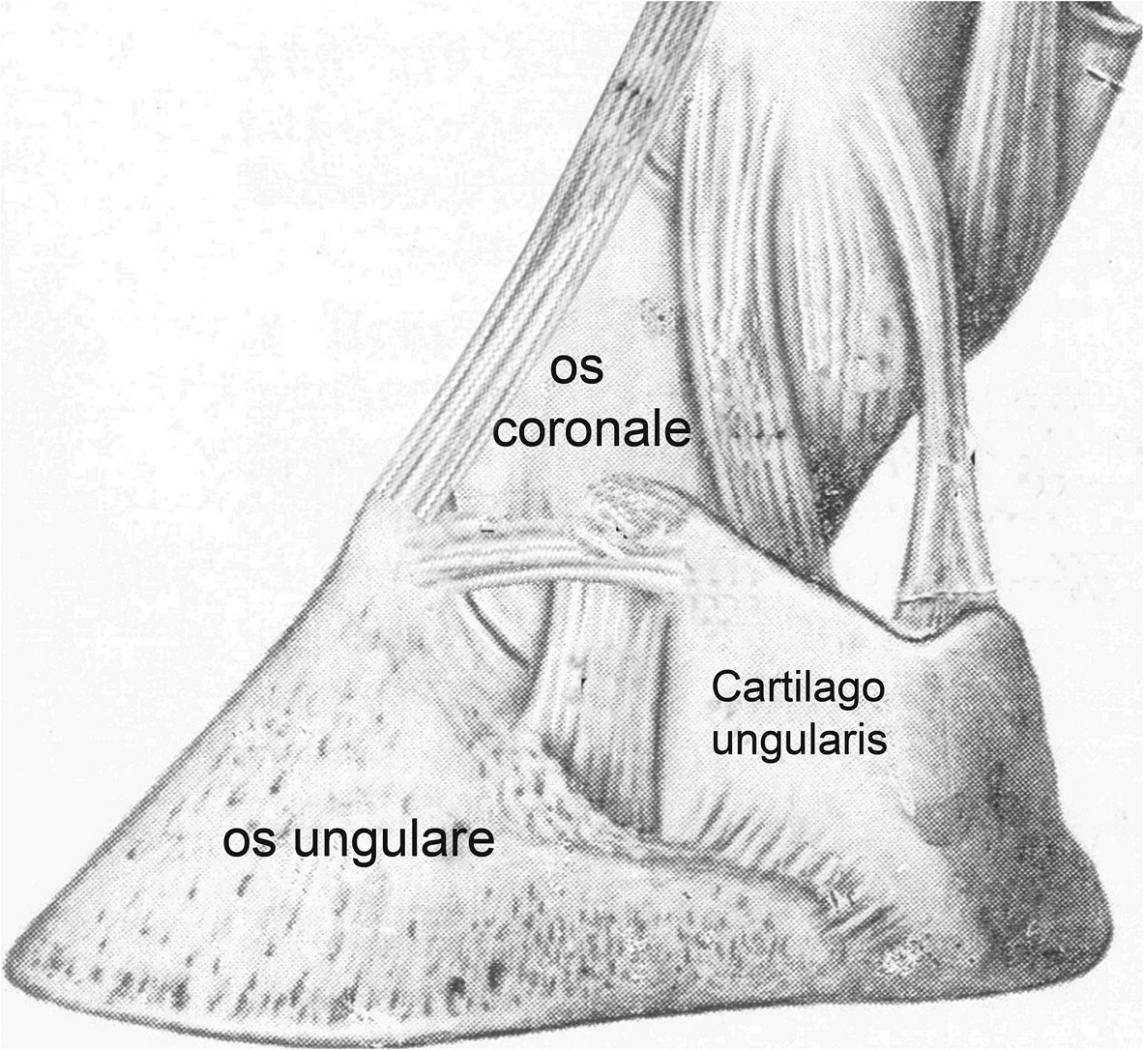
**Normal anatomy.** Illustration of *cartilago ungularis*.

Ossification of the ungular cartilages (OUC) (*cartilago ungularis medialis et lateralis*, Figure 1), which is synonymous with side bones, in the foot of the horse has been studied in Europe for over 170 years [1,2]. In 1906, Witte [2] reported a prevalence of 94% in 1100 draught horses which worked by walking and trotting on hard surfaces in cities. The high heritability of this condition is well known in different breeds [3-7] and traditional hoof examination by palpation has over time been complimented by radiography, scintigraphy and magnetic resonance imaging [7-11].

Early development of cartilage in the hoof was described by Bragulla [12]. The cartilages are hyaline at birth, but are gradually changed into a fibrous cartilage. During early life the ungular cartilages are supposed to adapt to the workload, i.e. bodyweight and conformation, resulting in more extensive cartilages in the front feet [13]. Suggested contributing factors for the development of OUC are hoof and body size, weight, workload and environment [5,14].

Different evaluation scales have been used in radiological studies of OUC in Ardenner horses [15], Finnhorses [8], Warmbloods [16] and Brazilian Showjumpers [7], but the long term impact of extensive ossification on performance has been poorly evaluated. Official Swedish statutes on horse breeding exclude stallions of all breeds if OUC is severe or present early in life [17]. Among Scandinavian cold-blooded trotters, a high grade of OUC (grade 4-5, using a 5 point scale) is still considered a pathological condition and at least one stallion every five years is excluded from breeding because of this condition. Furthermore, to be included in Swedish official gene banks, horses and their parents have to be free from all grades of OUC [18]. The associations for different cold-blooded breeds in Scandinavia have over the years used different strategies and different scales in control programs for OUC. However, to this date none of the control programs has been proven successful in this respect.

Bragulla [12] described the locations of ossification in foals and Lejune *et al.* [15] the development until 28 months of age in Ardenner horses, but later development of OUC over time has not been presented for any breed.

Our hypothesis was that the grade of OUC may increase up to 3 years of age life and remain stable thereafter.

## Methods

This project was approved by the Uppsala Animal Ethics Committee in 2006 (No. C88/6).

Front hooves from a total of 649 Swedish and Norwegian cold-blooded trotters, 293 mares and 356 males, were evaluated by radiography regarding ossification of the ungular cartilages of the distal phalanx. For 197 mares and 211 males, all born 1995, the radiographs were taken at 2.5 ± 0.25 years in connection with the study reported by Holm *et al.* [19]. Twelve stallions were radiographed in connection with pre-breeding evaluation (mean age 5, median 4 years), and 229 (96 mares and 133 males) (mean age 5, median 6 years) in connection with gatherings of cold-blooded trotters through open invitations to horse owners living near three racetracks in the central third of Sweden. Depending on accessibility, 147 of the horses were re-examined 3-13 years (mean age 9, median 8 years) after the first occasion. For comparison, the first 62 horses were examined blind by both palpation and radiology, later only by radiology since palpation proved to be a very uncertain method of identifying horses with OUC and of separating different grades of OUC. For age distribution, see Table 1.

**Table 1:** **Age distribution of included horses**

**Age**	**Number of horses**	**Number of horses**
Age year	At first examination	At second examination
1	18	
1.5	27	
2	4	
2.5	445	
3	10	
3.5	29	1
4	32	
4.5	3	1
5	15	
6	21	
7	9	3
8	9	4
9	3	6
10	5	70
11	4	3
12	2	1
13	2	2
14	2	16
15	3	14
16	1	6
17	1	4
18	1	1
19	1	5
20		2
21	1	3
22		1
23		1
24		1
25	1	
28		1
29		1
Total	649	147

**Figure 2:**
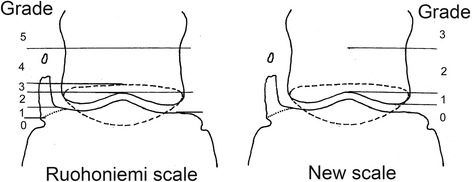
**OUC grading systems.** Grading of ossification according to the Ruohoniemi *et al*.^8^ scale (RS) and a new scale (NS) developed in this study. Left side shows moderate ossification with separate center of ossification and the dotted line is the navicular bone. Grading according to Ruohoniemi et al. Grade 0 No ossification, inclination sagittal. Grade 1 Ossification maximum to a level of palmar distal phalanx joint space. Grade 2: To a level of proximal palmar distal phalanx joint space. Grade 3: To a level of proximal border of navicular bone (dotted line). Grade 4: Proximal navicular bone up to distal half of middle phalanx. Grade 5: Ossification over distal half of middle phalanx. New grading according to SLU (new): Grade 0: Ossification not extending proximal of distal middle phalanx. Grade 1: Ossification extending between distal middle phalanx to a level of proximal palmar distal phalanx joint space. Grade 2: To a level of distal half of middle phalanx. Grade 3: To a level over distal half of middle phalanx.

Horses were identified in Swedish and Norwegian sports data [20] through registration number identification. Radiographs were taken on non-sedated horses by different veterinarians at several different equine clinics and in field conditions in stables. Mud was brushed off distal legs and hooves but no further cleaning or preparation was made. A dorso-palmar view with horizontal beam was used, with the horse standing on the floor or with the front hooves on 4-8 cm thick wooden blocks. Depending on the facility where the radiographs were taken, the film focus distance varied between 90 and 125 cm and the exposure values between 70-75 kV and 3.2-5.0 mAs using a high performance generator and x-ray tube or a portable x-ray unit. For 95% of the horses, a conventional film and a cassette with intensifying screens were used, while digital radiography was used for the remaining 5%. All radiographs were evaluated blind by the same person (Dr. Wattle) using the scale of Ruohoniemi *et al.* [8] (RS) (Figure 2). By comparison of the 2 sets of radiographs, in some horses it was clear that because of variation in radiographic technique the grading scale was inaccurate for grades 0 to 3. Therefore a simpler scale was devised and all radiographs were reassessed using a 0 to 3 scale (NS) (Figure 2). Grades 2 and 3 were equivalent to Ruohoniemi *et al*’s grades 4 and 5, respectively. The cartilage with the highest grade of ossification, including both the left and right front feet, determined the total score for an individual horse.

Body size score (height + chest circumference at withers) of 100 horses, from both Sweden and Norway, was either measured by us using stick and tape at the second examination or, in some cases, taken from official pre-breeding examination data using same methods [20].

### Statistical methods

To study the relationship between age and grade of cartilage, some important statistical issues must be considered. Cartilages are measured on an ordinal scale, here using two scales from 0 to 3 and 0 to 5, respectively. Four measurements were made per horse, on the lateral and medial position of each front hoof. This suggests that ordinal regression models, such as ordinal logit or ordinal probit models may be appropriate [21]. Our hypothesis was that the grade of OUC may increase up to 3 years of age and remain rather stable after that age. A model that could account for this is a piecewise regression model [21,22]. In such models, the relationship between *y* and *x* is modeled as one linear regression model for *x ≤ c* and another linear regression model for *x > c*, where c is a parameter to be estimated from the data. Piecewise regression models have been found to be useful in modeling age-related biological variables, for example the level of serum sialic acid in women before and after the menopause [23].

The above model features were combined into an ordinal piecewise regression model, which was programmed using the NLMIXED procedure of the SAS package [24]. The code used is available as supplementary material on the journal’s website. Four different model versions were compared here, based on the Akaike Information Criterion (AIC) [25,26]. All models were of the ordinal regression type, but with different model assumptions:

1. A piecewise linear relationship between age and grade of OUC.
2. A linear relationship between age and grade of OUC.
3. A quadratic relationship between age and grade of OUC.
4. Age included as a classification variable in the model.

Generalized linear mixed (GLM) models [21,22] were used for the analyses of body size vs. highest grade of OUC in front feet.

## Results

Both grading scales resulted in consistent results in horses without OUC and with high grades of OUC. Comparison between the two scales used in this study is illustrated by cross-tabulation in Table 2. A more thorough statistical comparison of the scales was not made. The incidence of OUC was 37% (grade ≥1 NS) and 85% (grade ≥1 RS).

**Table 2:** **Cross-tabulation of used scales**

**New scale 1-3 Ruohoniemi scale 1-5**	**0**	**1**	**2**	**3**	**Total number of horses**
**0**	100	0	0	0	100
**1**	206	2	0	0	208
**2**	99	94	3	0	196
**3**	2	21	33	0	56
**4**	1	3	44	1	49
**5**	0	0	1	36	37
Total	408	120	81	37	646

Cross-tabulation of the relationship between the Ruohoniemi scale and the new scale for assessing grade of OUC. Three missing values; one due to missing identification and the second and third due to non-complete sets of radiographs.

Even though many OUC became broader over the years, the breakpoint for the end of development of grades of OUC occurs close to 3 years of age (2.83 years, SE 0.1885). Before the age of 3, the slope of the linear predictor was significantly greater than zero (*P* = 0.0025), i.e. the estimated level of OUC increased. After this age, the slope was close to zero but not significant (*P* = 0.3084), which suggests that the level was rather stable. In re-examined horses 3 years and older at the first examination, i.e. 52 individuals, an increase in grade of OUC was seen in only five horses using the new scale, but only one of these five horses went from grade 0 to having OUC, i.e. from grade 0 to grade 1. The remaining four had OUC already at the first examination. In terms of individual cartilages, an increase in grade of OUC was seen in seven out of 208 cartilages in these 52 horses.

**Figure 3:**
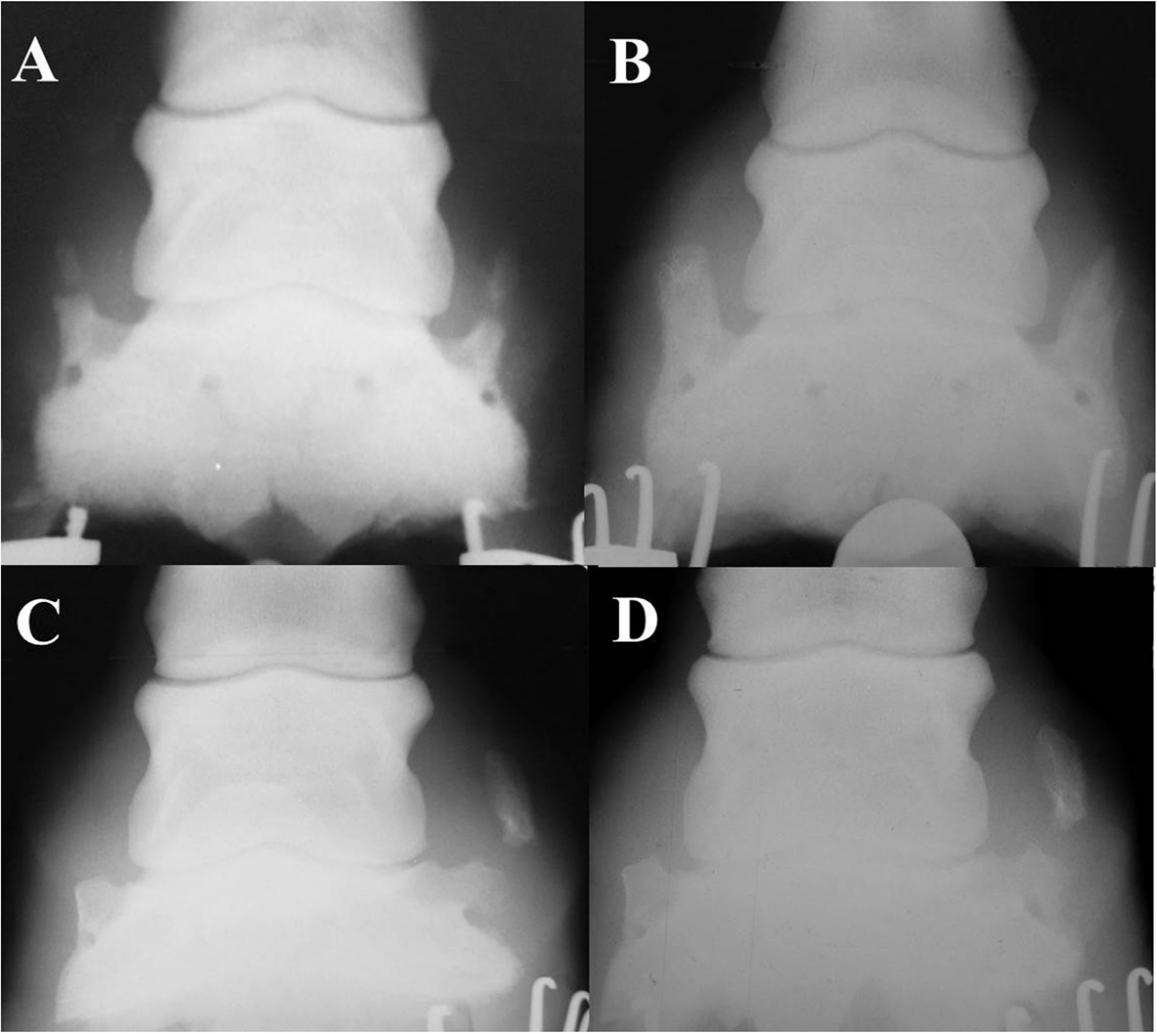
**Re-radiographed hooves.** All radiographs have lateral to the right. **A**. Radiographs of the left front hoof of a 2.5 year old cold-blooded mare with a highly ossified cartilage and separate centers of ossification in both lateral and medial sides. The grading’s were 4 and 5 with Ruohoniemi scale, plus 2 and 3 with the new scale. **B**. Same hoof eight years and 68 races later. Here ossification is more complete but the separate centers of ossification have not fused with the OUC originating from the base of the cartilage and there is no different OUC grading. **C**. Radiographs of left front hoof of a 6 year old unraced mare with OUC of grade 2 and 5, Ruohoniemi scale, and grade 0 and 3, new scale, medial and lateral respectively. **D**. Same hoof thirteen years and 58 races later, still with OUC of grade 2 and 5 using the Ruohoniemi scale but now with grade 1 and 3 according to the new scale.

Of the 649 horses examined, 43 had separate centers of ossification in a total of 88 cartilages. Eleven of these horses were also in the re-examined group and some of the centers had increased in size and some had remained the same, but none had fused with OUC originating from the base of the cartilage (Figure 3) and none had moved to a higher grade of OUC. We had to exclude evaluation of separate centers of ossification on two radiographs since radiological examination technique and artifacts, such as small amounts of mud on hooves, made it impossible to be sure if small separate centers of ossification was present or not.

The body size score varied from 325 to 374 cm with a mean of 348 cm. The 100 horses with complete scoring showed no significant correlation between body size and highest grade of OUC (*P* = 0.16).

The AIC value for statistical models 1-4 was 1074.4; 1087.1; 1082.1 and 1091.5, respectively. Since the AIC was smallest for model 1, the piecewise regression model, it displayed the best fit to the data. It was thus the model used for interpretation.

## Discussion

Our hypothesis that development of OUC, i.e. its proximal extension, occurs and stabilizes by 3 years of age was supported by the results. In horses 3 years and older at the first examination, the grade of OUC increased using the new scale in only 7 out of 208 cartilages examined twice. Some characteristics making the cold-blooded trotter a good model horse for OUC studies are its high performance potential and often long career, i.e. from 2 to 15 years of age. The majority of horses in this study had been in regular training since 1.5 or 2 years of age, and in high speed training (>7 m/s) on a weekly basis from 3-4 years of age. The 453 horses that had raced had completed a mean of 52 races (median 65) per horse. It therefore seems likely that the genotype of the horse and not the lifetime mechanical stress and loading of the hooves is the cause of OUC. By considering all horses, the breakpoint for development of OUC was calculated to be 2.8 ± 0.38 years using the 0-3 NS values as ordinal data in a suitable piecewise linear regression model. This is a reasonable result, since a moderate to high heritability of OUC has been reported in many studies [3-6].

Studies on fetal and early development of ungular cartilage and heritability in different breeds have added no information regarding development and clinical relevance [12,15]. At birth the cartilage is hyaline and during the postnatal period it adapts to workload, i.e. bodyweight and environmental factors, like all tissues of the hoof. The progression of OUC during the first years of life has been described for Ardenner horses [15], but it has not been shown whether it is possible to affect this progression during the first 1-2 years of life. The 2.8 year breakpoint calculated in this study is an age that corresponds well with the time in life that, according to our clinical experience, the distal limb has matured in cold-blooded trotters. However, size of the adult animal seems not to be an important factor in lighter cold-blooded horses, because there was no correlation between body size and grade or position of OUC in this study.

Using a grading system with many levels and the navicular bone as a point of reference increases the demands on the radiological examination technique. A few horses had an unexpected decreased grade of OUC at second blinded examination. In all radiographs used the grade of ossification was consistently determined and our only explanation for the unexpected decrease of OUC is how the horse loaded its hoof and the direction of the x-ray beam at the time of exposure. Our data was collected under field conditions and by different veterinarians using slightly different techniques. This is a limitation which in a few cases stopped us from making reliable detailed observations about separate centers of ossification and the character of existing fusion lines in radiographs. Naturally, the use of a two dimensional imaging technique preclude a full evaluation of a three dimensional development, i.e. ossification. Besides the obvious need to standardize the radiological examination technique, we believe that using our more forgiving new scale approach is beneficial in clinical practice, especially since our recent studies [27] indicated that OUC had no effect on performance in cold-blooded trotters and since field radiography, unfortunately, often is performed under non optimized circumstances.

Separate centers of ossification were found in 3% of cartilages examined and these showed only minor differences in range in re-examined hooves. Clinical experience shows that radiological examination technique and artifacts such as small amounts of mud on hooves might both affect the number of separate centers of ossification found in a population. However, the fact that we had this problem on two radiographs did not alter the result that presence of separate centers of ossification does not affect the performance of the horse [27].

Early studies describe OUC, mainly diagnosed through palpation, as a pathological condition causing lameness in heavy horse breeds [1,2]. However, clinical experiences over the years show that it is impossible to correlate the grade of OUC determined by palpation with the radiological findings. Thus, for the last 60 years [4,5] radiology has been the gold standard for evaluating OUC. This decreases the scientific value of many early studies on OUC in which the results were based on palpatory findings or post mortem examinations. Obviously, problems still remain regarding how to fully understand and interpret OUC using radiographs. Looking at possible causes of distal lameness, more recent studies are often performed on limited or selected groups of horses often with a history of low front limb lameness [10]. However, the same risk of over-interpretation still exists when not yet fully evaluated modern diagnostic tools such as MRI, scintigraphy and computed tomography are used when trying to solve the enigma of OUC as its pattern of development is closely similar to normal physiology and adaptation.

OUC is considered a pathological finding by several authors [28,29]. However, our results show that in cold-blooded trotters, OUC mainly develops before 3 years age, i.e. corresponding to a period of life when the rest of the distal limb matures. The early and heavy training of cold-blooded trotters generally exceeds the strain to which hooves in many other breeds are exposed. However, since detailed management and training data is not available for young horses in this study, effects of heritable vs. environmental factors early in life are still unknown. More detailed and specific diagnostic clinical research on young working horses (0-3 years of age) of different breeds is essential in order to fully understand the development of OUC.

## Conclusions

OUC is a condition that occurs and stabilizes early in life. For both clinical work and research a four grade system without relation to navicular bone is beneficial for the radiographic evaluation. Ordinal data from OUC can be used in a piecewise regression model. Results from this study can assist equine practitioners in understanding this condition in their clinical work and when devising breeding programs.
